# The associations between socioeconomic status and risk of *Staphylococcus aureus* bacteremia and subsequent endocarditis – a Danish nationwide cohort study

**DOI:** 10.1186/s12879-017-2691-3

**Published:** 2017-08-25

**Authors:** Louise Bruun Oestergaard, Michelle D. Schmiegelow, Niels Eske Bruun, Robert L. Skov, Andreas Petersen, Paal Skytt Andersen, Christian Torp-Pedersen

**Affiliations:** 1 The Institute of Health, Science and Technology, Aalborg University, Aalborg, Denmark; 2 Department of Cardiology, Copenhagen University Hospital, Gentofte; Kildegaards Vej 28, Post-635, 2900 Hellerup, Denmark; 3 The Department of Microbiology and Infection Control, Statens Serum Institut (SSI), Copenhagen, Denmark; 4 Clinical Institute, Aalborg University, Aalborg, Denmark

**Keywords:** *Staphylococcus aureus*, Social status, Young adults, Endocarditis, Risk factors, Education

## Abstract

**Background:**

*Staphylococcus aureus* bacteremia (SAB) is the leading cause of infective endocarditis in several countries. Since socioeconomic status (SES) is known to influence the risk of infectious diseases in general, we aimed to investigate the association between SES and SAB, and risk of subsequent endocarditis in a nationwide adult population.

**Methods:**

All Danish residents were consecutively included at age ≥ 30 years during 1996–2010. We obtained information on SES (highest attained educational level), comorbidities, and microbiologically verified SAB by cross-linking nationwide registries. The incidence rate ratios (IRRs) of SAB and later endocarditis were investigated using Poisson regression models adjusted for sex, age and year (reference = highest SES).

**Results:**

Our study population comprised 3,394,936 individuals (median age = 43.2 years). Over a median follow-up of 15.9 years, 13,181 individuals acquired SAB. SES was inversely associated with SAB acquisition, which declined with increasing age, e.g. in individuals with lowest SES, IRRs were 3.78 (95% confidence interval [CI] = 2.89–4.95) in age 30–50 years, 1.87 (CI = 1.60–2.18) in age > 50–70 years and 1.31 (CI = 1.11–1.54) in age > 70 years (interaction-*p* < 0.0001). Adjustment for comorbidities attenuated the IRRs, but the pattern persisted. No association between SES and endocarditis risk among patients with SAB was observed.

**Conclusions:**

Decreasing SES was associated with an increased risk of SAB, particularly in younger adults. SES was not associated with risk of subsequent endocarditis.

**Electronic supplementary material:**

The online version of this article (doi:10.1186/s12879-017-2691-3) contains supplementary material, which is available to authorized users.

## Background

*Staphylococcus aureus* bacteremia (SAB) is among the top ten causes of death in the Western world [1–3], and the high occurrence of multi-resistant *Staphylococcus aureus* limits the therapeutic options in many countries [4, 5]. While a number of risk factors for acquiring SAB have been explored [6–10], large-scale studies on the impact of socioeconomic status (SES) on risk of SAB is sparse. Furthermore, infective endocarditis is a frequent and severe complication to SAB [11], however it is as yet unexplored whether SES is associated with an increased risk of subsequent endocarditis in patients with SAB.

Studies have suggested a link between low SES and increased risk of infectious diseases in general [12–14] as well as increased all-cause mortality following bacteremia [15]. However, only one study (case-control) has examined the association between SES and risk of community-acquired bacteremia, with risk of SAB studied as a sub-analysis. In this study, the associated risk of SAB in individuals with low educational level was 4-fold increased compared with individuals with a high educational level [16].

Identification of individuals at high risk of SAB is crucial to improve prophylactic and treatment strategies. We therefore conducted a nationwide cohort study to assess the association between highest attained educational level and risk of acquiring primary SAB, and risk of subsequent endocarditis in an adult Danish population.

## Methods

### Data sources and definitions

To identify all Danish residents who were ≥30 years old during 1996–2010 nationwide registries were cross-linked at an individual level using the personal identification number assigned to all Danish residents at birth or immigration [17]. From *the Danish Civil Registration System* we identified all Danish residents who were ≥30 years old during 1996–2010, and further obtained information on vital statistics (date of birth/death and sex) and demographic data (immigration/emigration status).

#### Exposure, socioeconomic status (SES)

Since the majority of the Danish population would be fairly young at study inclusion and since educational level is known to reflect an individual’s future income [18], we defined SES as highest attained educational level at study entry categorized as follows: 1) basic school (primary, lower secondary); 2) upper secondary (general secondary, technical secondary; “high-school”); 3) vocational (e.g. electrician or chef); 4) short or medium length higher education (Academy Profession Degree, Professional Bachelor’s Degree, University Bachelor’s Degree; 2–4 years following upper secondary); 5) Master’s Degree or Ph.D.; as described elsewhere [19]. Data were achieved from *the Population’s Education Registry*, which contains information on each individual’s highest attained education for 96.4% of the Danish population between 15 and 69 years of age [20]. We excluded individuals with missing data on educational level, including all first generation immigrants.

For an additional analysis, we further obtained information about annual income in the year prior to study inclusion from *the Income Statistics registry on personal income* (e.g. income in year 2000 if included in 2001), which was categorized into quartiles using the highest quartile as reference [21].

#### Outcomes

The primary outcome was risk of microbiologically verified SAB, which was identified in *the National Danish Staphylococcus aureus Bacteremia Database* from the national reference laboratory at Statens Serum Institut. This registry was established as a collaboration between the Danish departments of Clinical Microbiology and Statens Serum Institut [22] in 1957. Isolates positive for *Staphylococcus aureus* are sent to Statens Serum Institut (on a voluntary level), which curates *the National Danish Staphylococcus aureus Bacteremia Database*. The registry comprises isolates and clinical data from >90% of all positive blood cultures in Denmark.

The secondary outcome was subsequent endocarditis in individuals diagnosed with SAB. We defined endocarditis following SAB as a diagnosis of endocarditis within 3 months after admission for SAB. All individuals with a diagnosis of endocarditis prior to SAB were excluded. The date of endocarditis was defined as the day of discharge. This information was obtained from *the Danish National Patient Registry* (ICD-10 codes = I33, I38) that holds dates and discharge diagnoses for all public and private hospital admissions in Denmark since 1977.

#### Comorbidities, prosthetic devices and surgical procedures

Information on medical conditions (e.g. diabetes, hypertension, cancer and valvular heart disease), prosthetic device implantations (e.g. pacemaker and hip prosthesis) and whether individuals had received chronic dialysis treatment or had surgery within 6 months prior to study inclusion were obtained from *the Danish National Patient Registry* [23]. Cancer at baseline was defined as any cancer diagnosis within 5 years prior to inclusion. Since diabetes and hypertension are primarily treated at the general practitioner, and thus not necessarily registered in *the Danish National Patient Registry* [24], we obtained information on these specific conditions by use of claimed prescriptions as recorded in *the National Prescription Registry* since 1995. All prescribed drugs are registered by the Anatomical Therapeutic Classification (ATC) with information on amount dispended and date of dispensing, and has been shown to be accurate [25]. Diabetes was defined as first claimed prescription for a glucose-lowering drug (ATC code = A10). We defined hypertension as claimed prescriptions for two different classes of antihypertensive drugs, as described in details previously [26].

### Statistical analyses

All Danish residents were consecutively included between January 1, 1996 and December 31, 2010, thus ensuring a follow-up period of minimum 1 year. Each individual was included in the study population at the last of the following events: January 1, 1996; the date the individual turned 30 years old; or the date of immigration to Denmark. The population was followed until primary admission for SAB, emigration, death, or December 31, 2011, whichever came first.

Baseline characteristics were defined as comorbidities prior to study inclusion and presented as median with interquartile range (IQR) for continuous variables, and as frequencies with percentages for categorical variables.

Multivariable Poisson regression models were used to assess the associations between SES and the risk of SAB. The Lexis macro was used for all analyses and included two time-scales: calendar time and age split in 1-year time bands after January 1, 1996. The expected numbers of events were calculated according to time at risk within each time band.

In our primary model we adjusted for age, sex and calendar year, and in a secondary model we additionally adjusted for the aforementioned comorbidities. Comorbidity status was updated time-dependently. For surgery during follow-up we introduced a “post surgery time period” where individuals were considered exposed to surgery from date of operation and 3 months onwards. Moreover, we examined whether the association between SES and risk of SAB differed by age or sex by use of statistical interaction tests, and stratified as appropriate.

Lastly, we explored the association between SES and risk of subsequent endocarditis in an analysis only including patients with SAB, and adjusted for age, sex and calendar year.

Results were reported with 95% confidence limits. Model assumptions (constant rate ratios for each interval) were tested by performing the same analyses using smaller time intervals (6 month intervals) and found valid unless otherwise indicated. Statistical calculations were performed using SAS for Windows, version 9.4 (SAS Institute Inc., Cary, NC).

### Additional and sensitivity analyses

Given the known close correlation between SES and comorbidities, we explored cumulative time exposed to selected comorbidities prone to increase the risk of SAB and endocarditis according to level of SES.

In an additional analysis we defined SES according to annual household income in the year prior to study inclusion, and explored the association between SES and risk of SAB adjusted for age, sex and calendar year.

Hypothetically, a lack of association between SES and risk of subsequent endocarditis in patients with SAB could appear due to a disproportional higher mortality among bacteremic patients from the lowest SES. Consequently, we examined cumulative incidences of subsequent endocarditis according to SES with death as competing risk. Finally, we examined potential differences in the association between SES and infective endocarditis in patients with SAB prior to and after 2007, since the Danish guidelines have recommended doctors to perform echocardiography in patients with SAB from this year [27].

### Ethics approval and consent to participate

This project was approved by the Danish Data Protection Agency with reference number 2007–58-015/ GEH-2014-018 I-Suite number: 02736. The clinical *Danish Staphylococcus aureus Database* was approved by The Danish Health and Medicines Authority (reference number 2007–54-0295). In Denmark, no ethics approval or informed consent is needed for retrospective register studies. All data are held by Statistics Denmark, which also has the administrative rights to the data and encrypts the identification number likewise in all datasets.

## Results

**Fig. 1:**
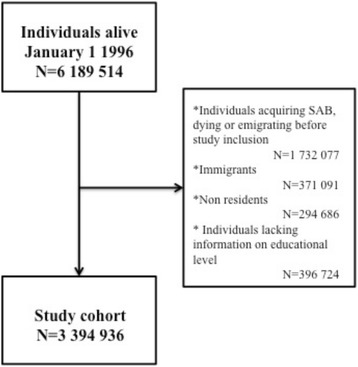
Flowchart. Flowchart illustrating the selection of the study cohort through nationwide registries

The study cohort comprised 3,394,936 individuals (Fig. 1) with a median age of 43.2 years (IQR = [30.0;56.0]). The distribution of men and women were overall equal (50.7% male), but across levels of SES, more men had vocational education and long higher education as highest attained education (Table 1). Individuals with the lowest SES were in general older, and rheumatic diseases, cancer, chronic obstructive pulmonary disorder and acute myocardial infarction were observed more frequently (Table 1).

**Table 1:** Baseline characteristics of the cohort stratified by socioeconomic status

Highest attained education	Basic school	Upper secondary	Vocational	Short/medium length higher education	Master’s Degree/Ph.D.
N (%)	1,230,283 (36.2)	161,342 (4.8)	1,291,659 (38.1)	553,451 (16.3)	158,201 (4.7)
Age (IQR)	50.9 (36.5–70.5)	30.0 (30.0–37.3)	41.4 (30.0–52.7)	38.9 (30.0–49.9)	38.3 (30.0–49.8)
Male (%)	543,959 (44.2)	80,772 (50.1)	732,954 (56.8)	236,722 (42.8)	100,711 (63.7)
Diabetes (%)	2384 (0.2)	212 (0.1)	1848 (0.1)	944 (0.2)	222 (0.1)
Hypertension (%)	843 (0.1)	255 (0.03)	1337 (0.2)	555 (0.1)	106 (0.1)
Periphere vascular disease (%)	8686 (0.7)	173 (0.1)	4840 (0.4)	1069 (0.2)	233 (0.2)
Acute myocardial infarction (%)	29,126 (2.4)	476 (0.3)	17,577 (1.4)	3862 (0.7)	1232 (0.8)
Valvular heart disease (%)	1754 (0.1)	83 (0.1)	1126 (0.1)	337 (0.1)	124 (0.1)
Chronic heart failure (%)	6555 (0.5)	153 (0.1)	3346 (0.3)	830 (0.2)	288 (0.2)
Mild liver disease (%)	6999 (0.6)	437 (0.3)	5411 (0.4)	1438 (0.3)	361 (0.2)
Severe liver disease (%)	2431 (0.2)	318 (0.2)	3103 (0.2)	1030 (0.2)	453 (0.3)
Acute renal failure (%)	1026 (0.1)	75 (0.1)	644 (0.1)	239 (0.1)	60 (0.1)
Chronical renal failure (%)	1014 (0.1)	79 (0.1)	715 (0.1)	220 (0.1)	59 (0.1)
Other renal disease (%)	1446 (0.1)	151 (0.1)	1397 (0.1)	542 (0.1)	152 (0.1)
Cancer (%)	24,504 (2.0)	1011 (0.6)	15,701 (1.2)	6374 (1.2)	1689 (1.1)
COPD (%)	24,422 (2.0)	475 (0.3)	10,137 (0.8)	2440 (0.4)	493 (0.3)
Psoriasis (%)	5400 (0.4)	311 (0.2)	3816 (0.3)	1368 (0.3)	317 (0.2)
Atopic dermatitis (%)	754 (0.1)	327 (0.2)	1278 (0.1)	858 (0.2)	247 (0.2)
Rheumatic disease (%)	44,246 (3.6)	1561 (1.0)	30,506 (2.4)	10,474 (1.9)	2326 (1.5)
HIV (%)	485 (0.04)	103 (0.1)	370 (0.03)	146 (0.03)	45 (0.03)
Surgical procedure^a^ (%)	6123 (0.5)	2363 (1.5)	11,576 (0.9)	6842 (1.2)	1448 (0.9)
Prosthetic device^b^ (%)					
• Cardiac	74 (0.01)	12 (0.01)	97 (0.01)	49 (0.01)	10 (0.01)
• Other	4755 (0.4)	1515 (0.9)	7742 (0.01)	3207 (0.6)	835 (0.5)
Chronic dialysis treatment^c^ (%)	33 (0.002)	7 (0.004)	18 (0.001)	10 (0.002)	-

Socioeconomic status is defined as highest educational level prior to study inclusion

COPD, chronic obstructive pulmonary disease; HIV, human immune deficiency virus

^a^Any surgical procedure within 6 months prior to study entry. ^b^Cardiac devices: Pacemaker, prosthetic heart valves (mechanic/biological), cardiovascular stents. Other devices: Hip and knee prosthesis, bone fracture treated with metal implants ^c^Dialysis treatment (irrespective of indication) within 6 months prior to study entry

**Fig. 2:**
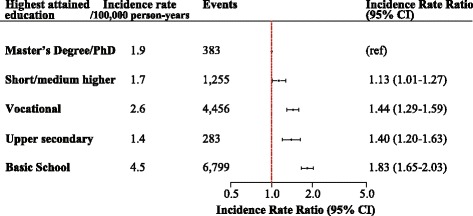
Incidence rate ratios of *Staphylococcus aureus* bacteremia according to socioeconomic status. Incidence rate ratios (IRR) of SAB according to SES adjusted for age, sex and calendar year (highest SES as reference)

**Fig. 3:**
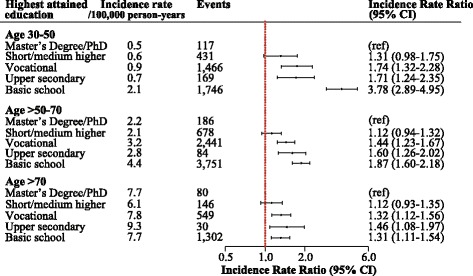
Incidence rate ratios of *Staphylococcus aureus* bacteremia according to socioeconomic status and stratified by age groups. Incidence rate ratios (IRR) of SAB according to SES stratified by age and adjusted for sex and calendar year (highest SES as reference)

During a median follow-up of 15.9 years (IQR = [10.7;16.0]) we identified 13,181 persons with a first hospitalization for SAB (62.9% male). We observed low SES to be associated with a nearly two-fold increased risk of SAB compared with the highest SES adjusted for age, calendar year and sex (Fig. 2). The association between SES and risk of SAB differed by age (p for interaction <0.0001), and we therefore stratified our results by age groups, i.e. 30–50 years (younger individuals), >50–70 years (older individuals) and >70 years (retired individuals) (Fig. 3).

In absolute terms, risks of SAB were lowest among young individuals aged 30–50 years (overall IR = 1.1/100,000 person-years) and increased with advancing age (overall IR = 3.5/100,000 person-years for 50–70 years-olds, and overall IR = 7.7/100,000 person-years for individuals >70 years). However, the highest relative risks of SAB were observed in the youngest age group with lowest educational level (basic school), and the relative risks decreased with advancing age (Fig. 3). Changing the age groups did not influence the pattern of the results. An additional figure displays these results [see Additional file 1].

In all models stratified by age, having a short/medium length higher education was not associated with increased risk of SAB compared with individuals with a Master’s Degree/Ph.D.

**Fig. 4:**
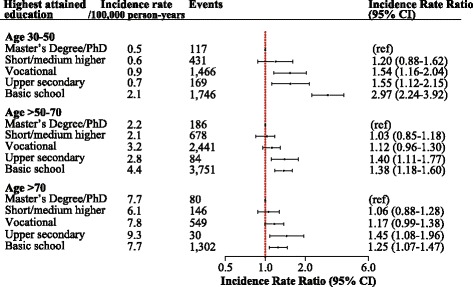
Fully adjusted model of incidence rate ratios of *Staphylococcus aureus* bacteremia according to socioeconomic status and stratified by age groups. The model is adjusted for sex, calendar year, diabetes, hypertension, acute myocardial infarction, hearth failure, valvular disease, cancer, periphery vascular disease, cerebrovascular disease, mild/severe liver disease, acute/chronic renal failure, chronic obstructive pulmonary disease, atopic dermatitis, psoriasis, rheumatic disorders, HIV, prosthetic devices, surgical procedures and dialysis treatment (highest SES as reference)

Adjusting for comorbidities, prosthetic devices and surgical procedures attenuated the inverse association between decreasing SES and increasing risk of SAB, but the pattern persisted (Fig. 4).

### Risk of subsequent endocarditis

**Fig. 5:**
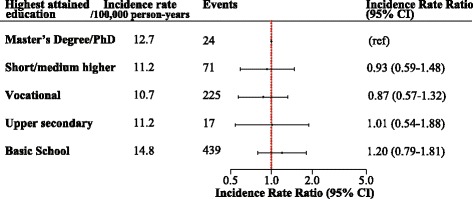
Incidence rate ratios of subsequent endocarditis in patients with *Staphylococcus aureus* bacteremia according to socioeconomic status. Incidence rate ratios (IRR) of subsequent endocarditis in 13,181 patients with SAB according to SES adjusted for age, sex and calendar year (highest SES as reference)

In patients with primary SAB (*n* = 13,181), 776 (5.9%) individuals were diagnosed with endocarditis within 3 months (IR of 13.1/100,000 person-years). Endocarditis was detected within the first week after hospitalization with SAB for more than 73% of the cases (*n* = 569). Nonetheless, SES was not associated with risk of subsequent endocarditis in patients diagnosed with SAB in a model adjusted for age, sex and calendar year (e.g. IRR of 1.20 [95% CI 0.79–1.81]) (Fig. 5). Additionally, the association between SES and endocarditis in individuals with SAB did not differ by age or sex, i.e. no interactions were observed between SES, and age or sex. Lastly, the lack of an association persisted in an analysis exploring the risk of endocarditis in SAB patients prior to and after 2007. An additional figure shows this in more detail [see Additional file 2].

### Other analyses

We further explored whether the cumulative follow-up time individuals were exposed to selected comorbidities prone to increase the risk of SAB and endocarditis (diabetes, cancer, dialysis, surgical procedures, valvular heart disease, pacemaker implantations and prosthetic heart valve replacement) differed across SES levels, and observed that individuals with basic school as highest attained education were more exposed to these comorbidities. An additional table shows these results [see Additional file 3].

**Fig. 6:**
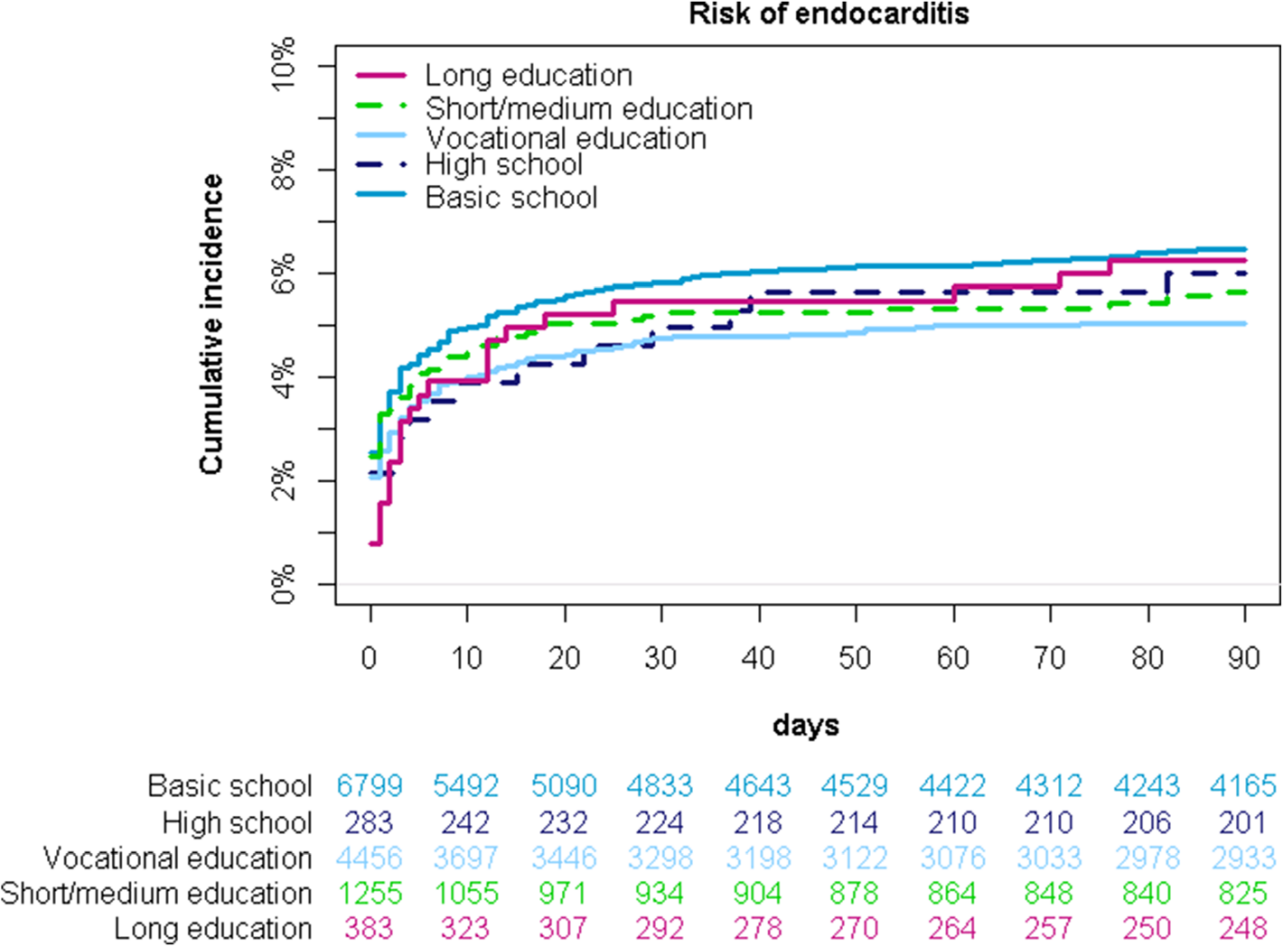
Cumulative incidence with death as competing risk. Cumulative incidence of endocarditis with death as competing risk in 13,181 SAB patients

Moreover, our finding of an inverse relation between SES and risk of SAB was unaltered when SES was defined by income in the year prior to study inclusion, but the relative risk estimates were higher as illustrated by an additional figure [see Additional file 4]. Lastly, the lack of an association between SES and subsequent endocarditis in patients with SAB did not appear to be explained by a higher mortality rate among individuals with low SES (Fig. 6).

## Discussion

In this nationwide cohort study of a population where everyone has free access to the healthcare system and has the right to treatment without a charge, we observed that declining level of SES was associated with an increasing risk of microbiologically verified SAB. Specifically, the associated risk of SAB in individuals with basic school as highest attained educational level was more than three-fold increased compared with individuals with long higher education. Additionally, the increased relative risks of SAB were most pronounced in the youngest age group and decreased with advancing age. Lastly, the level of SES did not appear to be associated with risk of subsequent endocarditis among patients diagnosed with SAB.

It has been demonstrated that SES may influence the subsequent risk of community-acquired bacteremia, and mortality after hospitalization with bacteremia, primarily due to a larger burden of drug-abuse and chronic diseases in individuals with low SES [15, 16]. In our cohort, individuals with low SES were more exposed to medical conditions, surgical procedures, dialysis treatment and implantations of both pacemakers and prosthetic heart valves. However, although the Danish registries do not hold information on all relevant risk factors the inequality in exposure to relevant risk factors only explained part of the inverse association between decreasing SES and increasing risk of SAB.

SAB is a rare condition in young adults [8], but our findings suggest that the majority of individuals, who acquire the disease in a fairly young age, are individuals with the lowest SES. Noteworthy, it appeared that the impact of SES decreased with advancing age, which may reflect that with advancing age, age-related comorbidities become more important for SAB risk than educational level.

Conversely, the definition of SES is more challenging in the eldest, as well as in the youngest part of the population with no definition of SES being perfect. Since the study population was fairly young at study inclusion, we found that educational level reflected SES better than annual income and have therefore chosen this parameter. Moreover, defining SES as annual income the year prior to study inclusion did not change the pattern of the results.

As the association between SES and risk of SAB varied by age we stratified the study population in to three age groups. We included the population at the age of 30 years and above to make sure that the majority had completed their education. Additionally, we defined the eldest age-group as >70 years to increase the likelihood that the patients were in fact retired, since it is becoming increasingly common for the elderly to remain on the labor market after the age of 65 years, which is the official age of retirement in Denmark. The study population with an age younger than 70 years was stratified into young (30–50 years) and older (>50–70 years) individuals at the labor market. In an additional analysis using an age cutoff corresponding to the Danish case-control study [16], the increased risk of SAB was still driven by the increased risk in the youngest subjects, which supports our age-categorization.

We chose to define SES as highest attained educational level at study entry since our study population was relatively young, thus income would be a less appropriate measure of SES. First, students are given subsistence (a fairly low amount of money to live on) during secondary and university education. Second, newly educated individuals start on a lower income right after end of their studies and third, retired individuals are given pensions, and thus all will be categorized as having a low SES, if defined by income. Furthermore, education is a fairly strong predictor of an individual’s future employment and thereby income, and education is unlikely to change after early adulthood [18]. Nonetheless, young individuals registered in the lower categories of educational attainment might in fact still be students in whom educational attainment does not accurately reflect SES, but this is probably not a substantial problem in our study, since we only included individuals’ ≥30 years of age. Additionally, defining SES according to level of income did not change the pattern of our findings.

*Staphylococcus aureus* is the leading cause of infective endocarditis in several countries [28, 29], but we did not find SES to be associated with subsequent risk of endocarditis among patients diagnosed with SAB, and the lack of an association did not appear to be caused by a higher mortality rate in individuals with the lowest SES. Importantly, we did not examine the overall association between SES and endocarditis, but rather if the level of SES influenced whether SAB was followed by endocarditis. The awareness of endocarditis increased noticeably during our study period, and since 2007, Danish guidelines have advocated doctors to perform both trans-thoracic echocardiography as well as trans-esophageal echocardiography in patients with SAB [27], and the guidelines endorsed by the European Society of Cardiology in 2009 [30] justify routine echocardiography in these patients. Hence, our findings may suggest that after 2007 patients with SAB are examined for endocarditis in accordance with guideline recommendations irrespective of their SES. Unfortunately, we could not investigate this further since information regarding echocardiography was not digitalized before late 2006 and echocardiographic data was not accessible through the nationwide registries. However, we analyzed potential differences in the association between SES and infective endocarditis in patients with SAB prior to and after 2007 and did not find any associations.

### Strengths and limitations

The present study offers several major strengths: we limited the risk of selection bias by including the entire Danish population and achieving data from nationwide registries; the completion of the Danish registers provides a minimal loss to follow-up; the coverage and validity of SES data is very high; and because SAB events were identified in the *Staphylococcus aureus* bacteremia Database, only microbiologically confirmed events were reported.

There are however some limitations, which merit comments. First of all, due to the register-based study design, we were not able to obtain adequate information on risk factors not systematically reported to the registries and only reported for a minor part of the population, such as information regarding intravenous drug abuse, dental procedures, smoking status or alcohol intake and comorbidities not in need of hospitalization or pharmacological treatment (e.g. diet-treated diabetes). Importantly, our main analysis of the association between SES and SAB adjusted for age, sex and calendar year and stratified by age groups was not subject to any of these limitations.

Secondly, a major limitation of this study lies in the registration of endocarditis, thus the ICD-10 codes for endocarditis in the Danish National Patient Registry, does not specify the microbiological etiology. Consequently, not all endocarditis events may be caused by *Staphylococcus aureus*. However, the majority of endocarditis events in these patients were diagnosed within a week after bacteremia diagnosis, and given the restrictive definition of endocarditis within a time limit of 3 months following SAB, this limitation is probably minor. We further excluded all patients with a diagnosis of endocarditis prior to SAB. The exclusion process may have omitted individuals whose SAB were in fact complicated by endocarditis, but in whom endocarditis was registered electronically prior to registration of SAB, resulting in possible underestimation of endocarditis events. Additionally, the number of endocarditis events following SAB could be underestimated due to the 3 months time period, which could explain why we observed a somewhat lower percentage of patients with subsequent endocarditis in our population compared with other studies [11, 31, 32]. It could also be explained by the fact that our study period includes 10 years where doctors did not use echocardiography routinely in patients hospitalized with SAB resulting in fewer endocarditis events. Nonetheless, the association appeared to be constant over time.

Identification of people at increased risk of SAB is an important step in preventing the disease. Our findings may contribute to increased awareness of the disease and the populations at risk, minimize doctor’s delay, and thereby improve the prognosis for these patients, for whom correct and timely treatment is crucial.

## Conclusion

We observed an inverse association between SES and risk of SAB with the highest relative risks observed in young adults (30–50 years old), which declined with advancing age, but was demonstrated irrespective of age. Additionally, the associations were only in part explained by comorbidities and risk factors prone to increase the risk of SAB. The level of SES did not influence the risk of subsequent endocarditis in patients with SAB.

## Additional files

Additional file 1: Incidence rate ratios of *Staphylococcus aureus* bacteremia according to SES. Incidence rate ratios (IRR) of *Staphylococcus aureus* bacteremia in accordance to SES stratified in two age groups (30–65 years and >65 years). The IRR are adjusted for calendar year, age and sex (highest SES used as reference). (EPS 137 kb)

Additional file 2: Incidence rate ratios of infective endocarditis in patients with *Staphylococcus aureus* bacteremia according to SES. Incidence rate ratios (IRR) of infective endocarditis in patients hospitalized with *Staphylococcus aureus* bacteremia prior to and after 2007 in accordance to SES. The IRR are adjusted for calendar year, age and sex (highest SES used as reference). (EPS 140 kb)

Additional file 3: Exposure time in person years for selected comorbidities stratified by SES. Exposure time for selected comorbidities stratified by SES (DOCX 79 kb)

Additional file 4: Incidence rate ratios of *Staphylococcus aureus* bacteremia according to income. Incidence rate ratios (IRR) of *Staphylococcus aureus* bacteremia according to income stratified by age and adjusted for calendar year, age and sex (highest quartile used as reference). (EPS 115 kb)

## Abbreviations

IE: Infective endocarditis
SAB: *Staphylococcus aureus* bacteremia
SES: Socioeconomic status

## References

[1] Schønheyder HC, Paul M (2013). Placing the burden of bacteraemia in perspective. Clin Microbiol Infect.

[2] Goto M, Al-Hasan MN (2013). Overall burden of bloodstream infection and nosocomial bloodstream infection in North America and Europe. Clin Microbiol Infect.

[3] Søgaard M, Nørgaard M, Dethlefsen C, Schønheyder HC (2011). Temporal changes in the incidence and 30-day mortality associated with bacteremia in hospitalized patients from 1992 through 2006: a population-based cohort study. Clin Infect Dis.

[4] Purcell K, Fergie J (2005). Epidemic of community-acquired methicillin-resistant *Staphylococcus Aureus* infections: a 14-year study at Driscoll Children’s hospital. Arch Pediatr Adolesc Med.

[5] Libert M, Elkholti M, Massaut J, Karmali R, Mascart G, Cherifi S (2008). Risk factors for meticillin resistance and outcome of *Staphylococcus Aureus* bloodstream infection in a Belgian university hospital. J Hosp Infect.

[6] Bassetti M, Trecarichi EM, Mesini A, Spanu T, Giacobbe DR, Rossi M (2012). Risk factors and mortality of healthcare-associated and community-acquired *Staphylococcus Aureus* bacteraemia. Clin Microbiol Infect.

[7] Jacobsson G, Dashti S, Wahlberg T, Andersson R (2007). The epidemiology of and risk factors for invasive *Staphylococcus Aureus* infections in western Sweden. Scand J Infect Dis.

[8] Laupland KB, Lyytikäinen O, Søgaard M, Kennedy KJ, Knudsen JD, Ostergaard C (2013). The changing epidemiology of *Staphylococcus Aureus* bloodstream infection: a multinational population-based surveillance study. Clin Microbiol Infect.

[9] Nielsen LH, Jensen-Fangel S, Benfield T, Skov R, Jespersen B, Larsen AR (2015). Risk and prognosis of *Staphylococcus Aureus* bacteremia among individuals with and without end-stage renal disease: a Danish, population-based cohort study. BMC Infect Dis.

[10] Thulstrup AM, Sørensen HT, Schønheyder HC, Møller JK, Tage-Jensen U (2000). Population-based study of the risk and short-term prognosis for bacteremia in patients with liver cirrhosis. Clin Infect Dis.

[11] Rasmussen RV, Høst U, Arpi M, Hassager C, Johansen HK, Korup E (2011). Prevalence of infective endocarditis in patients with *Staphylococcus Aureus* bacteraemia: the value of screening with echocardiography. Eur J Echocardiogr.

[12] Burton DC, Flannery B, Bennett NM, Farley MM, Gershman K, Harrison LH (2010). Socioeconomic and racial/ethnic disparities in the incidence of bacteremic pneumonia among US adults. Am J Public Health.

[13] Ravishankar A, Singh S, Rai S, Sharma N, Gupta S, Thawani R (2014). Socio-economic profile of patients with community-acquired skin and soft tissue infections in Delhi. Pathog Glob Health.

[14] Baker MG, Barnard LT, Kvalsvig A, Verrall A, Zhang J, Keall M (2012). Increasing incidence of serious infectious diseases and inequalities in New Zealand: a national epidemiological study. Lancet.

[15] Koch K, Nørgaard M, Schønheyder HC, Thomsen RW, Søgaard M (2013). Danish collaborative Bacteremia network. Effect of socioeconomic status on mortality after bacteremia in working-age patients. A Danish population-based cohort study. PLoS One.

[16] Koch K, Søgaard M, Nørgaard M, Thomsen RW, Schønheyder HC, Danish collaborative Bacteremia network. Socioeconomic inequalities in risk of hospitalization for community-acquired bacteremia: a Danish population-based case-control study. Am. J. Epidemiol. 2014;179:1096–1106. 10.1093/aje/kwu032 24682527

[17] Pedersen CB (2011). The Danish civil registration system. Scand J Public Health.

[18] Galobardes B, Lynch J, Smith GD (2007). Measuring socioeconomic position in health research. Br Med Bull.

[19] Jensen TB, Gerds TA, Grøn R, Bretler D-M, Schmiegelow MD, Andersson C (2013). Risk factors for venous thromboembolism during pregnancy. Pharmacoepidemiol Drug Saf.

[20] Jensen VM, Rasmussen AW (2011). Danish education registers. Scand J Public Health.

[21] Baadsgaard M, Quitzau J (2011). Danish registers on personal income and transfer payments. Scand J Public Health.

[22] SAB 2013 version 4 - *Staphylococcus aureus* bakteræmi rapport 2013.ashx [Internet]. [cited 2015 Apr 20]. Available from: http://www.ssi.dk/~/media/Indhold/DK%20-%20dansk/Smitteberedskab/Referencelaboratorier/Stafylokoklaboratoriet/Staphylococcus%20aureus%20bakter%C3%A6mi%20rapport%202013.ashx

[23] Lynge E, Sandegaard JL, Rebolj M (2011). The Danish National Patient Register. Scand J Public Health.

[24] Kildemoes HW, Sørensen HT, Hallas J (2011). The Danish National Prescription Registry. Scand J Public Health.

[25] Gaist D, Sørensen HT, Hallas J (1997). The Danish prescription registries. Dan Med Bull.

[26] Olesen JB, Lip GYH, Hansen ML, Hansen PR, Tolstrup JS, Lindhardsen J (2011). Validation of risk stratification schemes for predicting stroke and thromboembolism in patients with atrial fibrillation: nationwide cohort study. BMJ.

[27] Dansk Cardiologisk Selskab - Kliniske rapporter [Internet]. [cited 2016 Jan 17]. Available from: http://nbv.cardio.dk/endocarditis#afs7_10.

[28] Selton-Suty C, Célard M, Le Moing V, Doco-Lecompte T, Chirouze C, Iung B (2012). Preeminence of *Staphylococcus Aureus* in infective endocarditis: a 1-year population-based survey. Clin Infect Dis.

[29] Bergin SP, Holland TL, Fowler Jr V, Tong S. Bacteremia, Sepsis, and Infective Endocarditis Associated with *Staphylococcus aureus*. Curr Top Microbiol Immunol. 2015;1–34. doi:10.1007/82_2015_5001. 10.1007/82_2015_5001 26659121

[30] Habib G, Hoen B, Tornos P, Thuny F, Endorsed by the European Society of Clinical Microbiology and Infectious Diseases (ESCMID) and by the International Society of Chemotherapy (ISC) for Infection and Cancer, Authors/Task Force Members (2009). Guidelines on the prevention, diagnosis, and treatment of infective endocarditis (new version 2009): the task force on the prevention, diagnosis, and treatment of infective Endocarditis of the European Society of Cardiology (ESC). Eur Heart J.

[31] Le Moing V, Alla F, Doco-Lecompte T, Delahaye F, Piroth L, Chirouze C (2015). *Staphylococcus Aureus* bloodstream infection and Endocarditis - a prospective cohort study. PLoS One.

[32] Kaasch AJ, Barlow G, Edgeworth JD, Fowler VG, Hellmich M, Hopkins S (2014). *Staphylococcus Aureus* bloodstream infection: a pooled analysis of five prospective, observational studies. J Inf Secur.

