# Supplementary material for: The associations between socioeconomic status and risk of Staphylococcus aureus bacteremia and subsequent endocarditis – a Danish nationwide cohort study
Source: BMC Infect Dis. 2017 Aug 25;17:589. doi: 10.1186/s12879-017-2691-3 (PMC5574102; doi:10.1186/s12879-017-2691-3)
Supplement: Supplementary file 3 — Exposure time in person years for selected comorbidities stratified by SES. Exposure time for selected comorbidities stratified by SES (DOCX 79 kb) [file 12879_2017_2691_MOESM3_ESM.docx]

**Additional file 3**

| **Additional file 3:** Exposure time in person years for specific conditions prone to increase the risk of *Staphylococcus aureus* bacteremia and endocarditis in a cohort of 3,394,936 individuals | | | | | |
| --- | --- | --- | --- | --- | --- |
|  | **Basic school** | **Upper Secondary** | **Vocational** | **Short/medium higher education** | **Mater’s Degree/Ph.D.** |
| **Age**  30-50 years old  >50-70 years old  >70 years old | 22743438617  31363339711  21715179483 | 6997020640  1844507238  362800176 | 35595367426  33452771171  11246857130 | 16078016414  13911285996  3776566562 | 4467282878  3613609339  1139275852 |
| **Risk factors** |  |  |  |  |  |
| Diabetes | 2646438427 | 112571849 | 1825968144 | 526218146 | 137624183 |
| Cancer | 9060726338 | 477204668 | 7540242269 | 3078178737 | 848292354 |
| Valvular heart disease | 1065174399 | 32748577 | 690141081 | 232569192 | 70026321 |
| Dialysis | 199704640 | 8645842 | 155242362 | 42013217 | 10772595 |
| Surgical procedures | 13683970100 | 1166157447 | 12740981448 | 5104825116 | 1267748045 |
| Pacemaker/ICD | 647342801 | 22077715 | 490308772 | 154521994 | 56009721 |
| Prosthetic heart valves | 304414759 | 10874417 | 214979812 | 70173453 | 23526122 |
